# A Practical Treatise on Venereal Disease; or, Critical and Experimental Researches on Innoculation, Applied to the Study of These Affections; with a Therapeutic Summary and Special Formulary

**Published:** 1843-02-18

**Authors:** 


*A Practical Treatise on Venereal Disease; or, Critical and Experimental Researches on Innoculation, applied to the Study of these Affections; with a Therapeutic Summary and Special Formulary.* By Ph. Ricord, M. D., Surgeon of the Venereal Hospital at Paris, etc., etc. Translated from the French. By Henry Pilkington Drummond, M. D. Philadelphia: 1843. 8vo. pp. 256.

No surgeon has done more towards the elucidation of the nature and treatment of this horrible malady than Dr. Ricord. The practical results of his zeal and research have been an improved therapeutics, and a vast amelioration in the amount of suffering. The publication of the present translation will contribute to the further dissemination of sound views of practice and pathology.

